# Adult Stem Cell Therapies for Wound Healing: Biomaterials and Computational Models

**DOI:** 10.3389/fbioe.2015.00206

**Published:** 2016-01-11

**Authors:** Daniele Tartarini, Elisa Mele

**Affiliations:** ^1^Department of Mechanical Engineering, Insigneo Institute for in silico Medicine, University of Sheffield, Sheffield, UK; ^2^Department of Materials, Loughborough University, Loughborough, UK

**Keywords:** mesenchymal stem cells, adipose stem cells, wound healing, cell-based modeling approaches, FLAME, Chaste

## Abstract

The increased incidence of diabetes and tumors, associated with global demographic issues (aging and life styles), has pointed out the importance to develop new strategies for the effective management of skin wounds. Individuals affected by these diseases are in fact highly exposed to the risk of delayed healing of the injured tissue that typically leads to a pathological inflammatory state and consequently to chronic wounds. Therapies based on stem cells (SCs) have been proposed for the treatment of these wounds, thanks to the ability of SCs to self-renew and specifically differentiate in response to the target bimolecular environment. Here, we discuss how advanced biomedical devices can be developed by combining SCs with properly engineered biomaterials and computational models. Examples include composite skin substitutes and bioactive dressings with controlled porosity and surface topography for controlling the infiltration and differentiation of the cells. In this scenario, mathematical frameworks for the simulation of cell population growth can provide support for the design of bioconstructs, reducing the need of expensive, time-consuming, and ethically controversial animal experimentation.

## Introduction

Human skin is a large and complex organ that is designated to protect the body against environmental insults, and it acts as barrier against chemical, mechanical, and thermal stresses, infections, and dehydration (Martin, 1997). Thanks to the presence of specific receptors and terminations of the peripheral nervous system, the skin exerts also regulatory and sensory functions, including regulation of body temperature, touch, and pain perception (Lumpkin and Caterina, 2007). Injuries, diseases, or surgical procedures can compromise the integrity of this vital organ with the disruption of its physiologic condition and the consequent formation of wounds (Metcalfe and Ferguson, 2007; Shaw and Martin, 2009). Once the skin is wounded, a cascade of biological processes starts in order to restore the normal tissue anatomy and assure wound closure (Bielefeld et al., 2013). If the healing process is delayed or it fails, a state of pathologic inflammation is established, resulting in chronic wounds. Impaired healing is often associated with ischemia, diabetes mellitus, tumor, venous and pressure ulcers, severe infections, and it can be the cause of reduced quality of life, disability, and even death (Gurtner et al., 2008).

In recent years, diverse strategies have been developed to effectively manage and cure chronic wounds (Metcalfe and Ferguson, 2007). Among these, therapies based on stem cells (SCs) are attractive thanks to the unique ability of these cells to self-renew and differentiate into function-specific cellular phenotypes (Wong et al., 2012). In particular, two types of adult SCs are relevant for promoting skin regeneration: mesenchymal stem cells (MSCs) and adipose-derived stromal cells (ASCs). The aim of this review is to provide an overview of the recent advances in the wound management area with emphasis on how biomaterials and SCs (MSCs and ASCs) can be combined to produce advanced wound dressings; how mathematical models and computation frameworks can be advantageously exploited to better understand the mechanisms of tissue regeneration and to design more effective medical devices.

## Instructive Biomaterial-Based Scaffolds

The ultimate aim of bioconstructs for wound healing is to accelerate the skin repair by creating a favorable environment for cell proliferation and differentiation, and mimicking the physicochemical and mechanical properties of the skin (Shevchenko et al., 2010; Yildirimer et al., 2012). Ideal biomedical devices for wound management should be able to reduce inflammation and microbial invasion. They should effectively absorb exudates, promote gas permeability, and deliver functional biomacromolecules to the wound site. Attempts to produce these advanced devices have led to the combination of SCs with properly structured biomaterials. Examples include epidermal, dermal, and dermoepidermal (composite) skin substitutes that encapsulate SCs and bioactive dressings with controlled porosity and surface topography for enhanced cell infiltration and differentiation. As biomaterials, collagen and hyaluronic acid [the major components of the extracellular matrix (ECM)] together with fibrin (abundant in blood clots and naturally involved in wound healing) are widely used for their high biocompatibility, degradability, and ability to promote cell proliferation, migration, and differentiation (Hu et al., 2014). On the other hand, biocompatible and biodegradable synthetic polymers, such as polycaprolactone, polylactic acid, polyglycolic acid, poly(vinyl alcohol), poly(ethylene glycol), and polyurethanes, are of interest in wound care, because they can be easily processed and their properties (mechanical strength and degradation rate) can be controlled and engineered (Moura et al., 2013). Lastly, polysaccharides, such as chitosan and its derivatives, are used for their antimicrobial and homeostatic activity and ability to stimulate fibroblasts proliferation, tissue granulation, reepithelialization, and collagen deposition (Hu et al., 2014).

### Mesenchymal Stem Cells

Mesenchymal stem cells are multipotent SCs that can be isolated from bone marrow and other tissues, including adipose and nerve tissue, amniotic fluid, and dermis (Fu and Li, 2009). They are capable to repair not only mesenchymal tissues (bone, cartilage, muscle, marrow, tendon, and ligament) but also liver, heart, nervous tissue, and skin. Furthermore, MSCs exhibit site-specific differentiation, responding to environmental cues and adapting their functions to diverse biomolecular contexts (Jackson et al., 2012). MSCs are involved in nearly all of the wound healing phases, stimulating angiogenesis, reducing local inflammation, and promoting the formation of the extracellular matrix. MSCs exhibit also antimicrobial activity, through the secretion of antimicrobial proteins or immune-modulating factors (Isakson et al., 2015; Zahorec et al., 2015). Preclinical studies have demonstrated that the local injection of bone marrow-derived MSCs (BM-MSCs) into an incisional full-thickness wound strongly reduces the healing time, promoting angiogenesis, reepithelialization, and granulation (Wu et al., 2007; Chen et al., 2008). Accelerated wound closure of diabetic ulcers has been also shown in preclinical and early human trials when BM-MSCs are used, thanks the production of key cytokines and growth factors, and differentiation in keratinocytes and endothelial cells (Badiavas and Falanga, 2003; Falanga et al., 2007; Jackson et al., 2012; Isakson et al., 2015). However, as the delivery of MSCs through direct injection can induce rapid cell death, novel strategies based on the use of MSC-seeded scaffolding materials have been proposed with the aim to promote cell adhesion, proliferation, and migration.

Cell- and collagen-derived dermal equivalents (DEs) have been produced using human BM-MSCs and MSCs from umbilical cord’s Wharton Jelly (UC-MSCs) in coculture with the keratinocyte cell line HaCaT (Schneider et al., 2010). Differently from cell-based DEs (without collagen), the cells were distributed homogenously in the collagen-based DEs, spreading and migrating within the porous structure of the scaffold. Furthermore, ECM proteins and growth factors were highly expressed indicating that collagen-based DEs efficiently directed cell proliferation and ECM remodeling. BM-MSCs and skin-derived (SD) MSCs in combination with collagen-based dermal substitutes (Integra and Pelnac) have been used also for the treatment of full-thickness wounds (Shevchenko et al., 2010; Leonardi et al., 2012; da Silva Jeremias et al., 2014). Studies on a murine model highlighted that Integra was faster colonized in animals receiving MSCs than in control ones (no MSCs) because MSCs promoted cell migration to the wound site and vascularization of the scaffold mainly due to a paracrine mechanism. SD-MSCs well adhered and established cytoplasmic extensions within the matrices, maintaining their phenotypic profile and creating a three-dimensional (3D) cell culture. A recent study has investigated the temporal and spatial migration of MSCs *in vivo* through porous collagen scaffolds loaded with stromal cell-derived factor-1α, demonstrating that the chemotactic cue promoted the recruitment of MSCs to the injured area. Consequently, the enrichment of the wound site with MSCs facilitated the reepithelialization and neovascularization of the tissue (Chen et al., 2015).

Together with DEs, micro- or nanostructured scaffolds for MSC-based therapies have been developed. Composite nanofibrous substrates of collagen and poly(l-lactic acid-co-e-caprolactone) (PLLCL) have been produced by electrospinning and used to direct the epidermal differentiation of human BM-MSCs (Jin et al., 2011). The physical characteristics (size, network organization, and mechanical properties) of the nanofibers and the biochemical cues of collagen were exploited to recreate a fibrillary environment mimicking the native skin. BM-MSCs cultured on the collagen-PLLCL nanofibers exhibited an excellent proliferation rate and their fibroblastic morphology gradually progressed toward that one of epidermal cells. Electrospun nanofibers of collagen and poly (d,l)-lactic-co-glycolic acid (PLGA) containing BM-MSCs were instead proposed for the treatment of full-thickness skin wounds (Ma et al., 2011). The collagen-PLGA scaffolds were implanted *in vivo* and MSCs promoted collagen synthesis and reepithelialization of the insulted skin.

As proved by clinical trials, collagen- and fibrin-based biomedical devices combined with MSCs are particularly promising for non-healing and chronic wounds (Li et al., 2015). A study on 20 patients, whose non-healing wounds (burns, lower extremity ulcers, and decubitus ulcers) were treated with a collagen sponge impregnated with BM-MSCs (Yoshikawa et al., 2008), has showed complete recovery and regeneration of the native tissue for the majority of the cases. In another study, complete or significant closure of diabetic ulcers has been observed using fibrin glue and collagen matrix containing BM-MSCs (Ravari et al., 2011).

### Adipose Stem Cells

Multipotent SCs from the adipose tissue are clinically attractive because they can be easily extracted in large amounts and possess high recovery yield (Hassan et al., 2014). It have been demonstrated that ASCs enhance wound healing by differentiating into endogenous skin cells, enhancing epithelial migration and dermal fibroblast proliferation, promoting angiogenesis, secreting cytokines and growth factors (insulin-like growth factor, hepatocyte growth factor, vascular endothelial growth factor), and reducing scar formation.

Similarly to MSCs, ASCs are typically administered by direct injection or topically through gel matrices. However, these approaches are detrimental for cell survival, and hardly provide a microenvironment suitable for cell proliferation and differentiation. In order to achieve therapeutic efficacy, bilayer nanofibrous structures have been proposed for the delivery of ASCs (Pan et al., 2014). Electrospun fibers of poly(e-caprolactone-co-lactide)/poloxamer (PLCL/poloxamer) have been combined with a substrate of dextran and gelatin by mimicking the multilayer structure of the skin. While the electrospun scaffold provided mechanical support and protection of the injured area against external stresses, the hydrogel offered a physiological environment for ASCs proliferation. Nanofibers of polyvinyl alcohol (PVA), gelatin, and azide have been developed for directing the differentiation of ASCs to keratinocytes (Ravichandran et al., 2013). Cells grown on scaffolds functionalized with azine expressed keratin and filaggrin (markers of epidermal differentiation), acquiring the characteristic morphology of keratinocytes. Chitosan-electrospun mats reinforced with cellulose or chitin nanocrystals have been also proposed as highly biocompatible and non-cytotoxic scaffolds for ASCs proliferation (Naseri et al., 2014, 2015).

Together with electrospinning, freeze drying has been used as technology to create 3D porous constructs. Structures of poly(3-hydroxybutyrate-co-hydroxyvalerate) (PHBV) loaded with ASCs have been tested *in vivo*, demonstrating that the mechanical properties of the scaffolds were able to control contraction stresses during tissue repair, whereas ASCs enhanced granulation, reepithelialization, and vascularization (Zonari et al., 2015). Scarring was strongly reduced during healing and, after 28 days of treatment with PHBV/ASCs samples, the new-formed tissue was characterized by a well-organized dermal matrix with sebaceous glands and hair follicles.

Preclinical studies have demonstrated that ASCs combined with engineered scaffolds based on natural biomaterials, such as collagen and cellulose derivatives, have high potential therapeutic effects in wound healing, because they increase the epithelialization rate, granulation, and downregulate the inflammatory response (Hassan et al., 2014; Rodrigues et al., 2014).

## Computational Models

The variety and complexity of the biochemical and biophysical processes involved in tissue regeneration alongside their intrinsic multiscale nature highlight the need of computational models both to fully understand cell growth and to design efficient scaffolds and tissue substitutes (Langer and Vacanti, 1993; Hori et al., 2004; Byrne et al., 2007; O’Dea et al., 2012). Aspects to be considered are the timely release of growth factors and therapeutic agents and the controlled degradation of the scaffold during wound healing to allow cells proliferation; especially for *in vivo* tissue regeneration that is more efficient than replacement (Yildirimer et al., 2012; Yildirimer and Seifalian, 2014). Multiphase models have been used to describe these time-dependent processes *in vitro* in a perfusion bioreactor, with particular attention for the interplay between cell growth, access to nutrients, and scaffold degradation (O’Dea et al., 2013). Cell population and culture medium have been modeled as viscous fluids within the porous scaffold, while the scaffold and ECM have been treated as rigid porous materials. The model has predicted that scaffold and ECM heterogeneity impacts on the mechanical properties of the regenerated tissue with effects on the future success of the implant. Further computational methods have modeled cell spreading and tissue regeneration *in vitro* using porous scaffolds by considering transport and consumption of nutrients, ECM deposition, cell population dynamics, cell attachment, migration and intercellular interactions (Sengers et al., 2007; O’Dea et al., 2012; O’Dea et al., 2014; Yildirimer and Seifalian, 2014). The diffusion of nutrients, oxygen, and biochemical signals is mainly accounted in the models as advection–reaction–diffusion equations and depends on the type of bioreactor or scaffold used. Finite element methods (FEM) and computational fluid dynamics (CFD) models have been proposed to understand how the scaffold/bioreactor structure and porosity affect the distribution of nutrients and consequently the cellular growth rate (Olivares and Lacroix, 2013). Although comprehensive computational models specifically conceived for skin regeneration are unavailable to date, most of the already developed methodologies can provide insights in modeling skin and wound healing. In the following, we will focus on the computational models suitable for skin regeneration, in particular for cell population dynamics, human skin homeostasis, and growth factors interactions.

### Continuum and Individual-Based Models of Cell Populations

**Figure 1:**
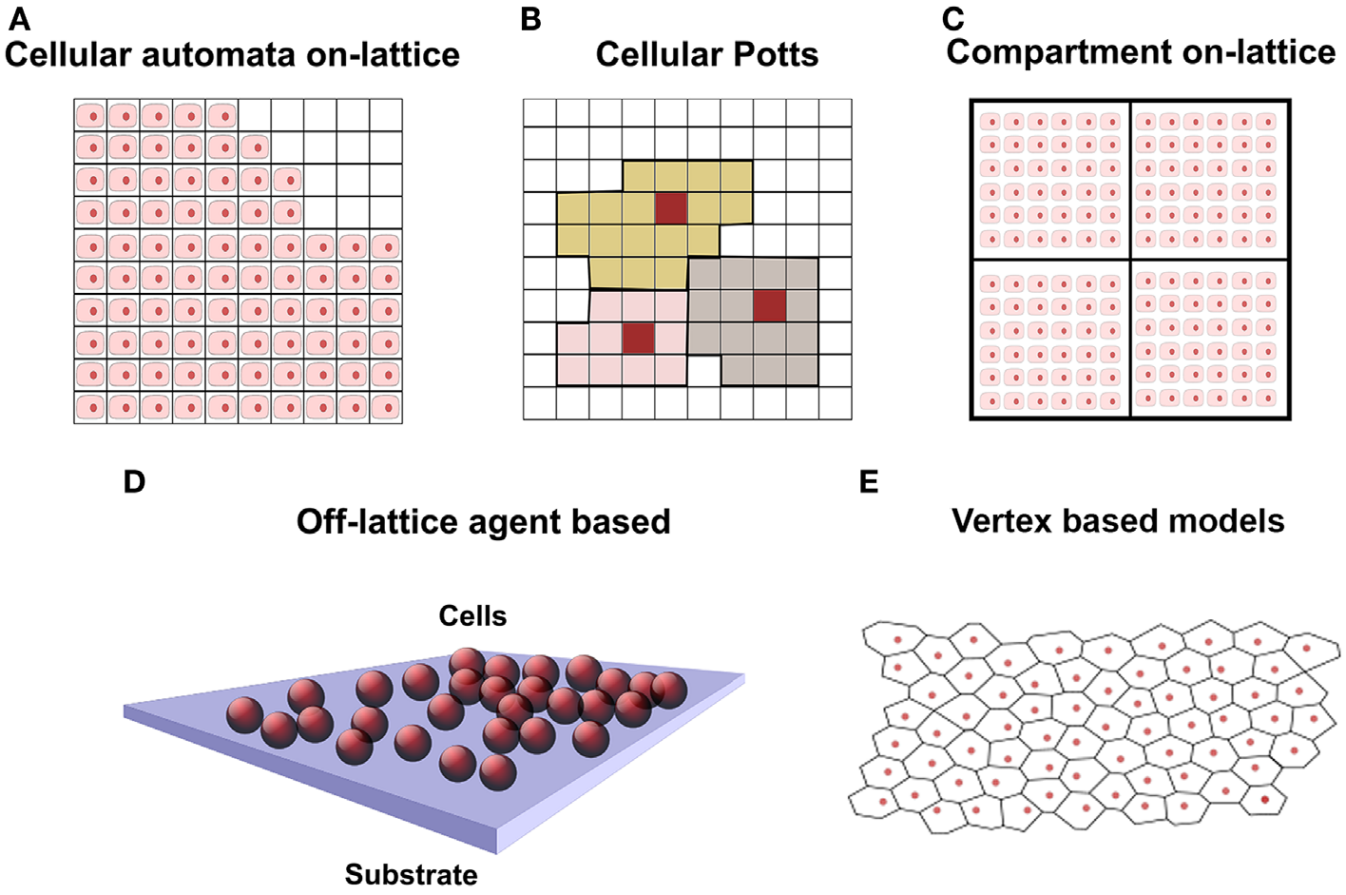
**Schematic representation of cell population in discrete models, where cells are represented in pink with nucleus in red**. **(A)** On-lattice approach: squared 2D lattice where each lattice element contains one single cell. At the top right, void locations are free to be occupied by daughter cells. **(B)** Cellular Potts model: squared lattice where each cell occupies several lattice elements. Cells are represented with different colors. **(C)** Compartmental model 2D: similar to squared lattice but having several cells per lattice element. **(D)** Off-lattice agent-based approach in 3D: cells are represented by spheres and are not constrained in a lattice. **(E)** Off-lattice vertex-based 2D: cell surface delimited by polyhedral vertices of a Voronoi tessellation.

Models for cell population growth are classified by the underlying mathematical approach: continuum, individual-based, and hybrid (O’Dea et al., 2012; Van Liedekerke et al., 2015). The debate on the adoption of a continuum versus an individual-based approach is extensively addressed in O’Dea et al. (2012). Continuum approaches are mainly based on multiphase or mixture theory that describes systems made of several interacting constituents, like a biological tissue (O’Dea et al., 2012): different cell types, ECM, and interstitial fluid. These systems can be represented as a mixture of continua, occupying the same spatial region, whose interactions are described through force balance equations and constitutive relations. Continuum models can be solved efficiently via FEM but cell properties are spatially averaged. On the other hand, individual-based or agent-based models (ABMs) are preferred when the number of initial cells is relatively small, which is the typical scenario for scaffolds seeded with SCs, and when subcellular phenomena need to be addressed, like cell signaling, cell cycle, cell–cell interaction, space occupancy. ABMs allow to explicitly express and study single cell behavior, signaling, proliferation, and movement (Youssef et al., 2007). Cell behavior is modeled through simple rules that take into account the cell cycle, the status of neighbors, and the space occupancy. These models are divided in on-lattice and off-lattice (Figure 1), depending on whether the cells are constrained in a lattice or are free to move in the space. Cellular automata (CA) models (Figure 1A) represent a cell as a lattice site with a fixed volume; biological and physical interactions are encoded in each cell as rules. Cell division, migration, and death are accounted shifting neighbors within an interaction radius. In Cellular Potts models (CPM), Figure 1B, a cell occupies several contiguous lattice sites (Graner and Glazier, 1992). Migration, growth, and shape change are modeled with a Markov chain Monte Carlo method and only favorable energetic configurations are accounted. In Figure 1C, lattice sites are compartments hosting several cells. This approach is similar to CA, but single cell position is not computed.

In off-lattice cell-centered ABM models (Figure 1D), cells are free to move in the space (also called lattice-free approach) and are modeled as spheres or ellipsoids. Nevertheless, when it is necessary to account the influence of mechanical forces on the cells (cell–scaffold and cell–ECM interaction), they are modeled as a deformable objects (Byrne and Drasdo, 2009). Off-lattice cell-centered ABM models have been used to model 2D *in vitro* epithelial tissues (Walker et al., 2004; Sun et al., 2007) and skin tissue growth in 3D (Adra et al., 2010); in both cases, the Flexible Large-scale Agent Modeling Environment (FLAME) computational framework for agent-based simulation has been used (Richmond et al., 2010). Further work on the lattice-free cell-centered approach has been done to take into account cell–cell and cell–environment interactions (Meineke et al., 2001; van Leeuwen et al., 2009). The models considered the cells connected through linear over-damped springs, and they have been used to simulate epithelial growth of the intestinal crypt. Voronoi polyhedra have been used (Figure 1E) to model a more realistic cell shape and contact surface in dense tissues with many neighboring cells, like in epithelia and skin (Fletcher et al., 2013). These models are implemented in the Cancer, Heart and Soft Tissue Environment (CHASTE) framework. The features and limitations of the computational models described above are summarized in Table 1. The computational power required for simulations depends on the model and the number of cells involved and code parallelization improves performances (Richmond et al., 2010; Harvey et al., 2015).

**Table 1:** **Comparison of cell population models (Van Liedekerke et al., 2015)**.

Computational models		

	Characteristics	Limitations
**On-lattice models**		
• Individual representation of cells		
• Precise cell position		
• Simulation of cell movement, division, and death		
(A) Cellular automata models	• Large-scale simulations	• Inappropriate description of cell mechanics and adhesion
	• Efficient parameter sensitivity	• Fixed cell size
(B) Cell Potts models	• Flexible and extensible framework	• Sensitivity analysis limited by computational complexity
	• High cell density can be simulated	• Physics partially represented
(C) Compartmental models	• Cell position resolved at the lattice compartment level	• Scale linked to lattice size
	• Efficient parameter sensitivity analysis	• Representation of physical interaction with energy function
**Off-lattice models**		
• Individual representation of cells		
• Physical laws directly represented		
• Variable cell size		
(D) Center-based models (CBM) with spherical cells	• Equation of motion is intuitive and extendable	• Cell-cell forces are pairwise and can generate artifacts
	• Effective code parallelization	• Large simulations (over 10^6^ cells) limited by computational time
(E) Vertex-based models	• Suitable for highly packed populations	• Computational complexity limits simulations to thousands of cells
	• Forces and mechanical stresses at subcellular level can be modeled	

Hybrid cell-center-continuous approaches have also been proposed and implemented (Cheng et al., 2009; Chung et al., 2010). They are based on a CA model for the cell cycle, cell proliferation, migration and collision, and on reaction–diffusion equation for nutrient concentration. These models have been used to investigate a typical condition of bioreactors where tissue regeneration is slowed by nutrient limitations, allowing the identification of more effective seeding strategies.

### Computational Models for Human Epidermis

As discussed previously, only few works have reported on computational models for skin and wound healing (*in virtuo* analysis), due to the complexity of this biological process. The epidermis studies available are based on ABM models with an initial population of SCs. Cells are generally approximated with spheres of 10 μm, and their behavioral rules are taken from literature or experimental data. ABMs have been used to investigate the organization and self-regulation of keratinocytes (Sun et al., 2007), the role of growth factors on cell–cell and cell–ECM interaction (Adra et al., 2010), the effect of the presence of fibroblasts on the expansion rate of keratinocyte colony (Sun et al., 2008), the spatio-temporal dynamics of epidermis homeostasis under normal and pathological conditions (Zhang et al., 2014), and the importance of SCs in long-term skin epithelium regeneration and homeostasis (Li et al., 2013). ABMs have been used to study the behavior *in vitro* of normal human keratinocytes under varying extracellular calcium concentrations, observing that the cell–substrate contact is crucial in the self-organization of the colony and that rapid wound closure is promoted in a low calcium media (Sun et al., 2007; Smallwood, 2011). A multiscale integrated model of human epidermis have been developed coupling ABM (through FLAME) with the expression and signaling of growth factors for specific subcellular mechanisms through COmplex PAthway SImulator (COPASI) (Hoops et al., 2006). FLAME has been also used to predict the dynamics of cell colonies over 3 years comparing different hypotheses of SC generation of epithelium (Li et al., 2013). The ABM models allow to explore alternative hypothesis about skin structure and dynamics over different conditions in timeframes longer than those feasible *in vitro* and in different regimes of nutrients or biochemical signals. ABM simulations of epithelial wounds made with FLAME can efficiently exploit parallel computational architectures and using GPUs obtain nearly real-time results (Richmond et al., 2010).

## Future Directions

Differently from bone tissue engineering where the understanding of bone structure, biomechanics, and tissue formation relies on a highly cross-disciplinary research (biomaterial engineering, biology, and computer science), the current state of the art of skin regeneration for wound healing is still sector-based. On one hand, advanced biomedical dressings have been developed using different classes of biomaterials and SCs; on the other hand, computational modeling has not yet been completely exploited to study skin growth and cell–biomaterial interaction. The complexity of the biological phenomena involved hardly permits the existence of a one-fits-all computational framework. Currently, the most mature frameworks supporting the research in this area (open source and supporting the main operating systems) are CHASTE for multiscale and multiphase problems (Mirams et al., 2013), CompuCell3D for multicellular organisms (morphogenesis) (Izaguirre et al., 2004), and FLAME for generic agent-based systems. Tools based on FEM also exist: FEniCS (Logg et al., 2012), ANSYS, and Abaqus. Theoretical and computational models can provide detailed information of physical and biological entities within the evolving/healing tissue that are not easily accessible with experimental studies: fluid and mechanical stress, cell density, and nutrient levels. Nevertheless, their prediction power at systems biology level is strictly linked to robust validation against biological models (Smallwood et al., 2004). The use of these technologies to predict the behavior of cells during wound closure and the role played by the dressing is fundamental to progress in this area, allowing the reduction of animal tests.

## Author Contributions

DT and EM conceived and wrote the main manuscript text, according to their competency.

## Conflict of Interest Statement

The authors declare that the research was conducted in the absence of any commercial or financial relationships that could be construed as a potential conflict of interest.
